# Supplementary material for: Dual engineering of thermodynamics and kinetics in covalent organic frameworks for separation
Source: Nat Commun. 2026 Mar 10;17:3896. doi: 10.1038/s41467-026-70311-8 (PMC13125621; doi:10.1038/s41467-026-70311-8)
Supplement: Supplementary file 1 — Supplementary Information [file 41467_2026_70311_MOESM1_ESM.pdf]

Supplementary information

Dual engineering of thermodynamics and kinetics in covalent organic frameworks for separation

Zi-Rui Rao (饶梓睿)[1,2], Xu-Qin Ran (冉旭芹)[1,3], Zhi-Quan Li (李志泉)[2], Yanlong Chen (陈彦龙)[4], Xiu-Ping Yan (严秀平)[1,2] and Hai-Long Qian (钱海龙) [1,2*]

[1] *State Key Laboratory of Food Science and Resources, Jiangnan University, Wuxi 214122, China*

[2] *Institute of Analytical Food Safety, School of Food Science and Technology, Jiangnan University, Wuxi 214122, China*

[3] *Department of Light Chemical Engineering, Jiangnan University, Wuxi 214122, PR China*

[4] *School of Pharmaceutical Sciences, Guangzhou University of Chinese Medicine, Guangzhou, Guangdong, 510006, China.*

Corresponding authors

Email: hlqian@jiangnan.edu.cn

Table of Contents

Supplementary Methods

Supplementary Figures S1-S31

Supplementary Tables S1-S13

Supplementary Methods

Materials and chemicals

All chemical reagents used are of analytical grade or higher. 1,3,5-Tris(p-formylphenyl)benzene (TpB), were bought from Jilin Chinese Academy of Science-Yanshen Technology Co., Ltd. (Jilin, China). Mesitylene, methanol (MeOH), tetrahydrofuran (THF), acetonitrile, n-hexane, ethyl acetate (EtOAc), HCl and NaOH were purchased from Sinopharm Chemical Reagent Co., Ltd. (Shanghai,China). $Fe(OTf)_2$, CF_3SO_2Na, glacial acetic acid, 1,2-dichlorobenzene (o-DCB), ethanol (EtOH), dimethyl sulfoxide (DMSO) and n-butanol (n-BuOH) were bought from Aladdin Chemical Co., Ltd. (Shanghai, China). p-Phenylenediamine (Pa) and (3-Aminopropyl)triethoxysilane were bought from Shanghai Titan Technology Co., Ltd. (Shanghai, China). Ultrapure water was obtained from Wahaha Foods Co., Ltd. (Shanghai, China). Fused silica capillary (0.53 mm i.d.) was purchased from Yongnian Optic Fiber Plant (Hebei, China).

Instruments

Thermogravimetric analysis (TGA) was inspected using a Q500 TG analyzer (Mettler-Toledo, Switzerland) under a N_2 environment at a heating rate of 10 °C min^{-1} from 30 °C to 800 °C. X-ray diffraction spectrometry (XRD) patterns were recorded on a D2 PHASER (BRUKER AXS GMBH, Germany) using CuKα radiation. N_2 adsorption experiments were performed on Autosorb-iQ (Quantachrome, USA) at 77 K. Fourier transform-infrared (FT-IR) spectra were obtained on an IS10 FT-IR spectrophotometer (Nicolet, USA). Scanning electron microscope (SEM) images were recorded on an SU8100 scanning electron microscope (Rigaku, Japan). Transmission electron microscopy (TEM) and Cryo-TEM images were recorded on a JEM-2100 PLUS (JEOL, Japan) and Thermo Scientific Glacios (Thermo Fisher Scientific, Czech),

respectively. X-ray photoelectron spectroscopy (XPS) measurements were performed using a METTLER TGA2 system equipped with a monochromatic X-ray source. AVANCE NEO (Bruker, Germany) spectrometer was used for producing ^{1}H (600 MHz) and ^{13}C (150 MHz) nuclear magnetic resonance (NMR) spectra, and ^{13}C solid-state nuclear magnetic resonance (SNMR) spectrum were acquired on a JNM-ECZ series NMR spectrometer (JEOL, Japan). QTRAP 4500 mass spectrometer (AB SCIEX, USA) equipped with a Nanospray II Source (P/N #1004600, AB SCIEX) was used to acquire MS data.

Synthesis of Pa-F

Pa (54.0 mg), $Fe(OTf)_2$ (17.7 mg), CF_3SO_2Na (117.0 mg) acetonitrile (5 mL) was mixed in 15 mL vial and flushed with N_2 gas three times[1]. Then vial was closed with Teflon and Parafilm, then placed under LED light (440 nm) and stirred at room temperature for 24 h. The product was purified by column chromatography (n-hexane/EtOAc = 95:5) using 60-200 mesh silica gel to afford 46% isolated yield of desired product Pa-F. ^{1}H and ^{13}C NMR (DMSO-d6, δ) spectra were showed in Figure S1 and S2. MS for C7H7F3N2: 176.0645 (calculated, 176.0561).

Preparation of NH_2- and TpB-modified capillaries

Fused silica capillaries (30 m × 0.53 mm) were pretreated with 1 mol L^{-1} NaOH solution, water, 1 mol L^{-1} HCl, and water in sequence. Then a mixture of MeOH/APTES (1:1, v/v) was filled into the pretreated capillaries. After sealing both ends, the capillaries were incubated at 70 °C for 24 h, then rinsed with EtOH, and dried with N_2 at 120 °C to obtain NH_2-modified capillaries. Dimethyl sulfoxide solution of TFPB (29.3 mg in 3 mL DMSO) was further injected into NH_2-modified capillaries. The capillaries were sealed, incubate at 60 °C for 2 hours, then rinsed with methanol to remove the residue, and finally dried with nitrogen flow at 120 °C for 2 h to obtain

TpB-modified capillaries.

Distribution coefficient (*K*)

$$K = k \times \beta = \frac{t - t_0}{t_0} \times \frac{V_m}{V_s} \quad (1)$$

Where k is capacity factor, t is retention time, t_0 column void time, β is phase ratio, V_m is volume of mobile phase, V_s is volume of stationary phase.

Column efficiency

Column efficiency is represented with theoretical plate number (*N*).

$$N = 5.54 \times (\frac{t}{W_{1/2}})^2 \quad (2)$$

where $W_{1/2}$ is full width at half maximum.

Resolution (*R*)

$$R = \frac{2(t_2 - t_1)}{W_2 + W_1} \quad (3)$$

where t_2 is the retention time of the second chromatographic peak, t_1 is the retention time of the first chromatographic peak, W_2 is the peak width of the second chromatographic peak, and W_1 is the peak width of the first chromatographic peak.

Thermodynamic parameters

Enthalpy change (ΔH) and entropy change (ΔS) for the chromatographic separation process were calculated according to the van't Hoff equation:

$$lnK = -\Delta H/RT + \Delta S/R \quad (4)$$

where K is distribution coefficient, R is gas constant, T is absolute temperature.

Van Deemter equation

$$H = A + B/u + C \times u \quad (5)$$

where H is theoretical plate height, A is eddy diffusion term, B is longitudinal diffusion term, C is mass transfer resistance term, u is linear velocity of mobile phase.

Eddy diffusion can be ignored for open tubular capillary columns, so the equation can be simplified as:

$$H = \frac{B}{u} + C \times u \tag{6}$$

$$H/u = \frac{B}{u^2} + C \tag{7}$$

Mass transfer resistance term (C)

$$C = C_s + C_m \tag{8}$$

Where C_s and C_m is the mass transfer resistance of stationary phase and mobile phase, respectively.

The C_m remains approximately constant when using the same analyte and mobile phase. C_s can be further determined by the following equation.

$$C_s = \frac{\omega_s d_f^2}{D_s} \tag{9}$$

where ω_s is a constant related to the structural properties of the stationary phase, d_f is the thickness of stationary phase, and D_s is the diffusion coefficient of the solute in the mobile phase.

Density functional theory

Density functional theory (DFT) calculations were performed using Gaussian 09. Molecular structures including model representative unit of COFs, analytes and COF-analyte composites were fully optimized with B3LYP density functional and 6-31+G (d) basis set[2-6]. The bonding energies (BE) were calculated as follows:

$$BE = E_{composite} - E_{COF} - E_{Analyte} \tag{10}$$

where $E_{composite}$, E_{COF}, and $E_{Analyte}$represent the energies calculated by DFT for the COF-analyte composites, representative unit of COFs, and analytes, respectively.

The Independent Gradient Model of the Hartree-Fock analysis (IGMH) was applied to visualize and explore the intermolecular interactions. The wave function file generated from the DFT single-point energy calculation was inputted in Multiwfn 3.8 program for an IGMH analysis (sub-option: 18, built-in feature: 200). The resulting data was subsequently visualized using the VMD 1.9.3 software.

Molecular dynamics simulation

All molecular dynamics (MD) simulations were performed using the CP2K software package. A simulation box was constructed with four COF layers to represent the solid TpBPa-F. In contrast, the hollow TpBPa-F model was created by computationally removing the central two layers from the original four COF layers simulation box. Then, 35 molecules of *o*-, *m*-, and *p*-CA were introduced on the feed side of both membrane models. When reaching dynamic equilibrium at 50 ps, the mean square displacement (MSD) of the three CA isomers along the z-direction (the direction perpendicular to the membrane surface) was calculated, and their self-diffusion coefficients on STpBPa-F and HTpBPa-F were subsequently derived from these MSD data.

Supplementary Figures

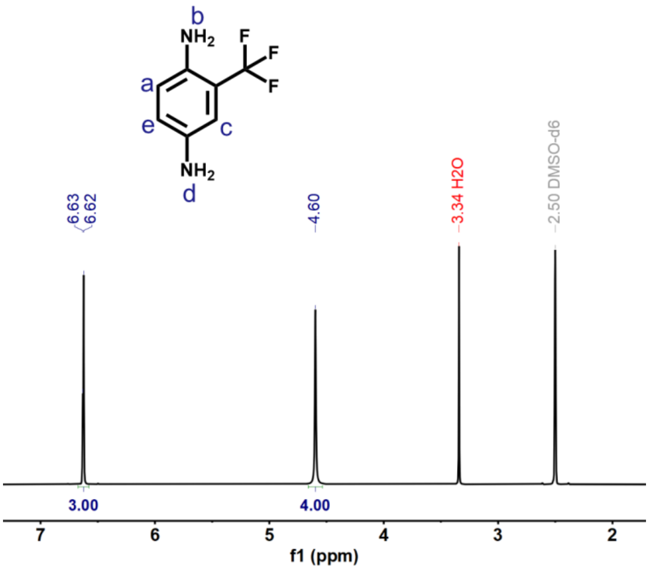

Supplementary Fig. S1 ^{1}H NMR spectrum of Pa-F.

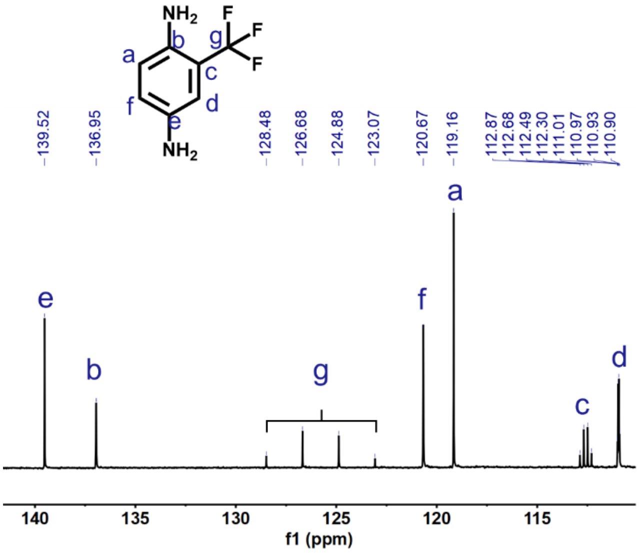

Supplementary Fig. S2 ^{13}C NMR spectrum of Pa-F.

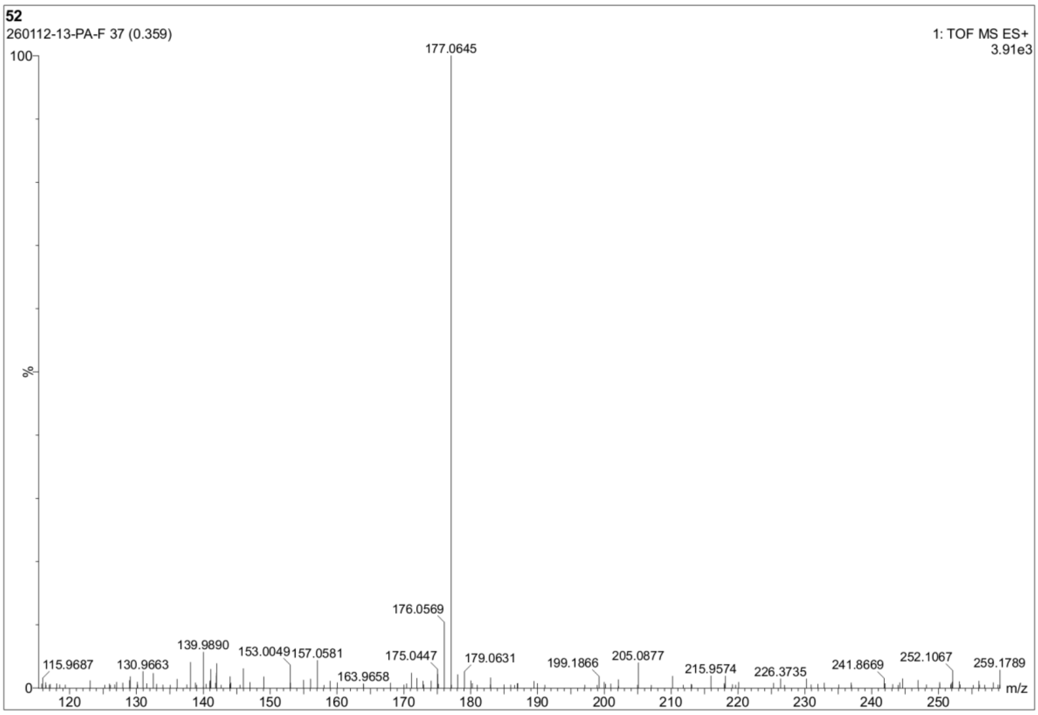

Supplementary Fig. S3 Mass spectrum of Pa-F.

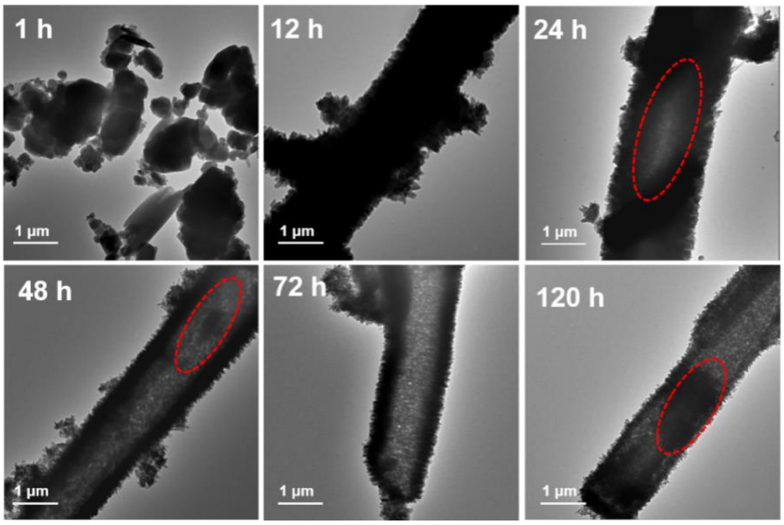

Supplementary Fig. S4 TEM images of TpBPa-F synthesized with different reaction time.

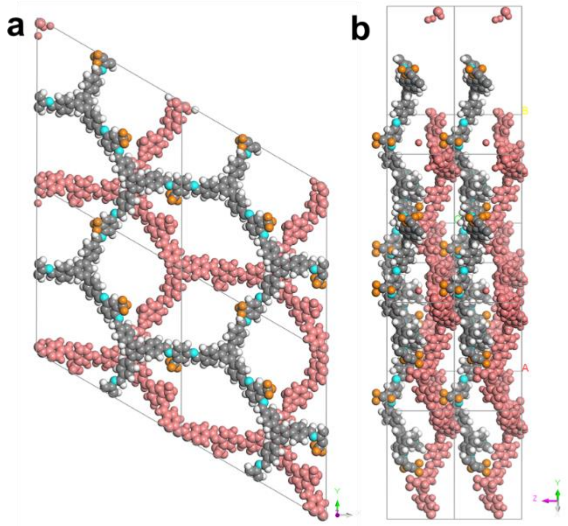

Supplementary Fig. S5 (a) Front and (b) side view of AB stacking unit cell of HTpBPa-F.

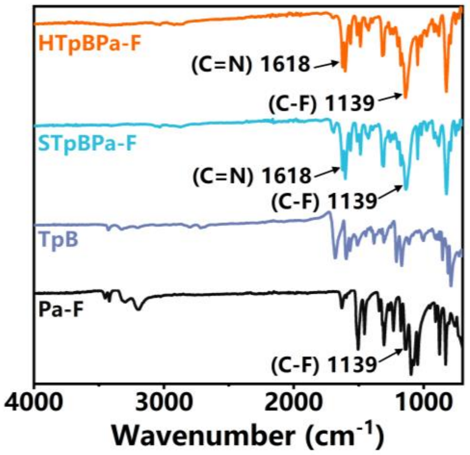

Supplementary Fig. S6 FT-IR spectra of Pa-F, TpB, STpBPa-F and HTpBPa-F.

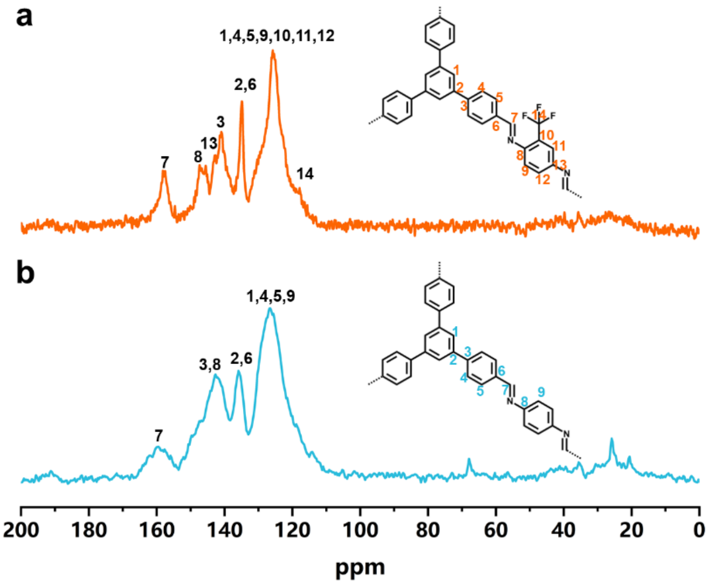

Supplementary Fig. S7 ^{13}C SNMR spectra of STpBPa and HTpBPa-F.

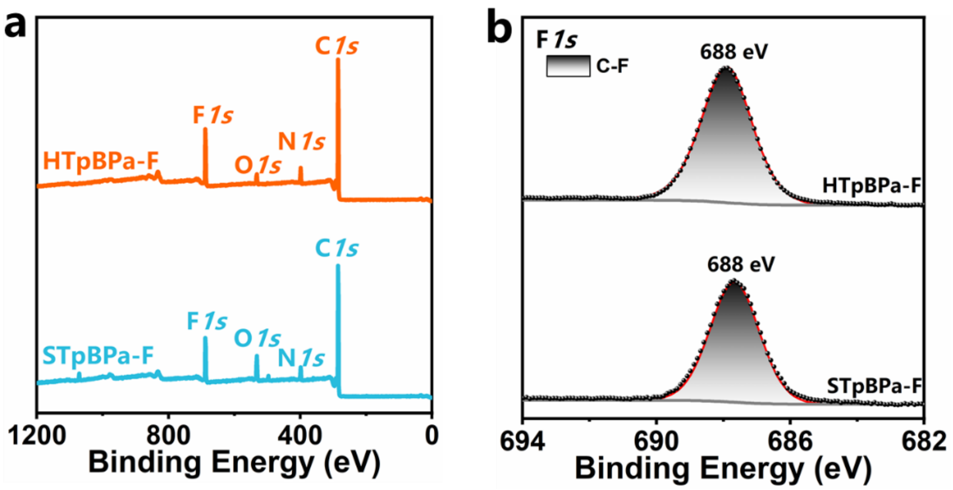

Supplementary Fig. S8 (a) XPS full spectrum of HTpBPa-F and STpBPa-F. (b) F *1s* XPS spectra of HTpBPa-F and STpBPa-F.

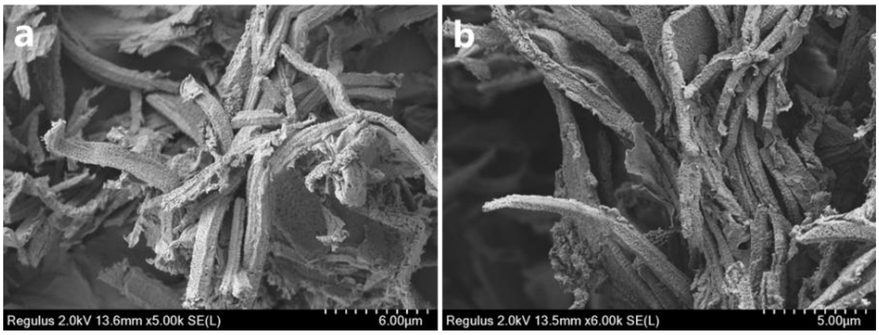

Supplementary Fig. S9 SEM images of (a) HTpBPa-F. (b) STpBPa-F.

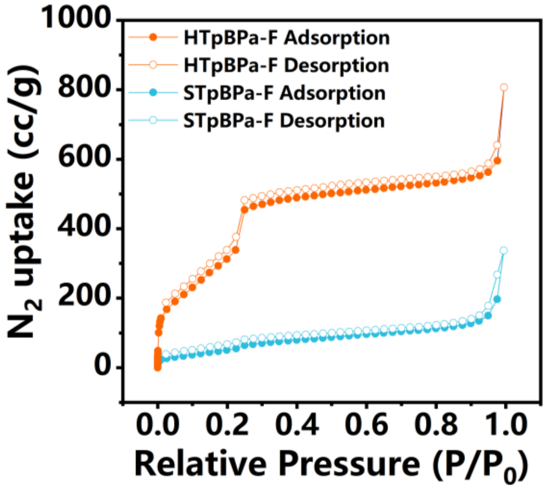

Supplementary Fig. S10 Nitrogen adsorption-desorption isotherms of HTpBPa-F and STpBPa-F.

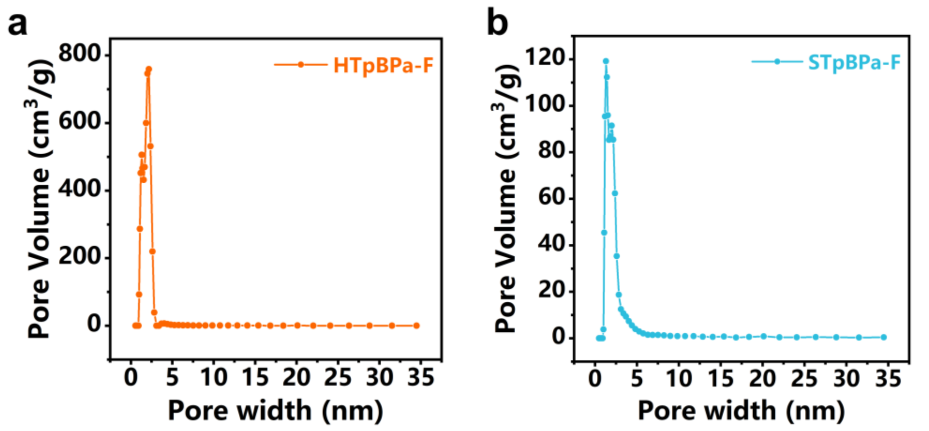

Supplementary Fig. S11 Pore size distribution of (a) HTpBPa-F and STpBPa-F.

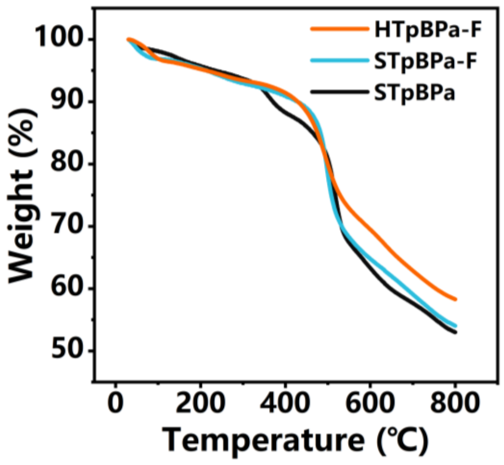

Supplementary Fig. S12 TGA curves of HTpBPa-F, STpBPa-F and STpBPa.

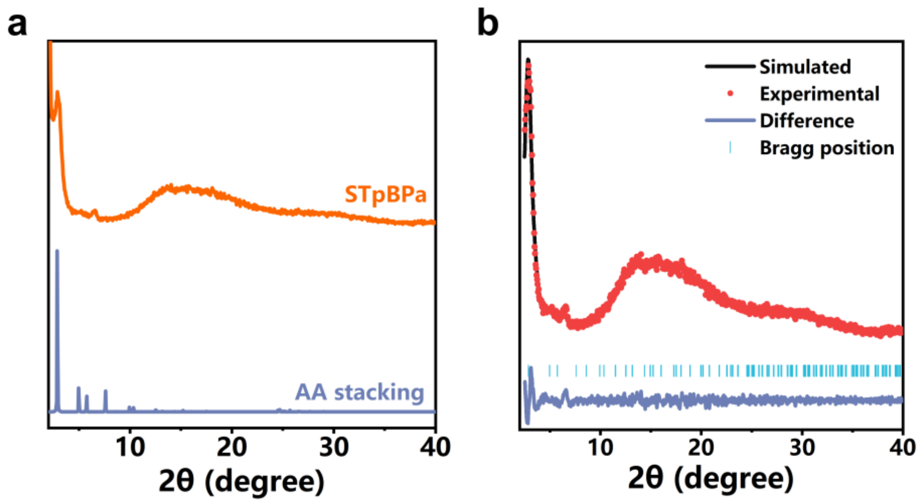

Supplementary Fig. S13 (a) Experimental and simulated PXRD patterns of STpBPa. (b) Pawley refinement results of simulated STpBPa (Rwp 5.47% and Rp 4.21%).

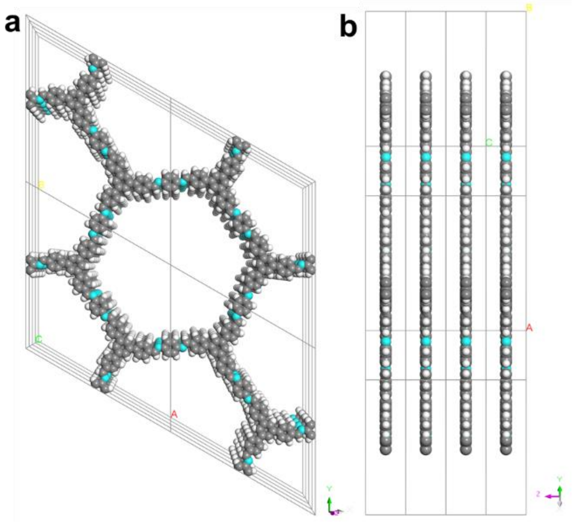

Supplementary Fig. S14 (a) Front and (b) side view of AA stacking unit cell of STpBPa.

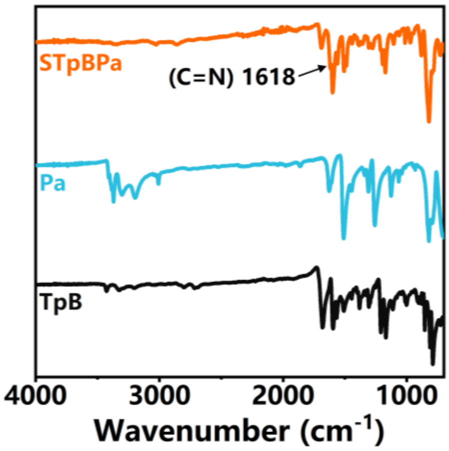

Supplementary Fig. S15 FT-IR spectra of TpB, Pa and STpBPa.

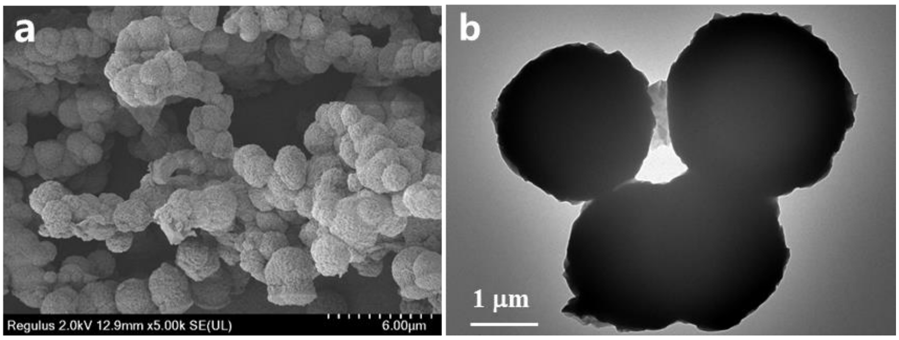

Supplementary Fig. S16 SEM and TEM images of STpBPa.

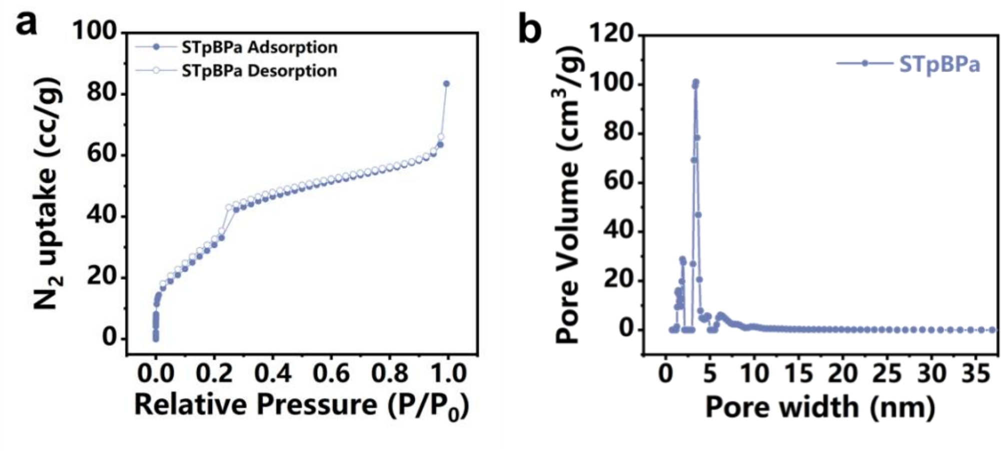

Supplementary Fig. S17 (a) Nitrogen adsorption-desorption isotherms of STpBPa. (b) Pore size distribution of STpBPa.

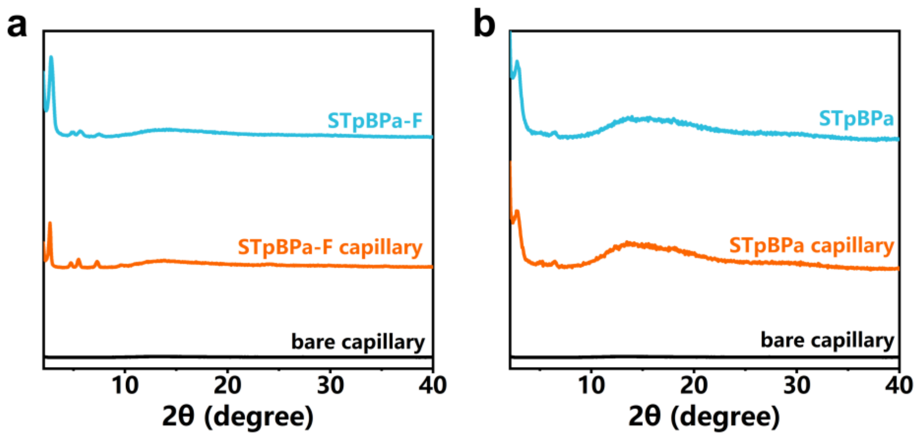

Supplementary Fig. S18 (a) PXRD patterns of bare capillary, STpBPa-F capillary and STpBPa-F. (b) PXRD patterns of bare capillary, STpBPa capillary and STpBPa.

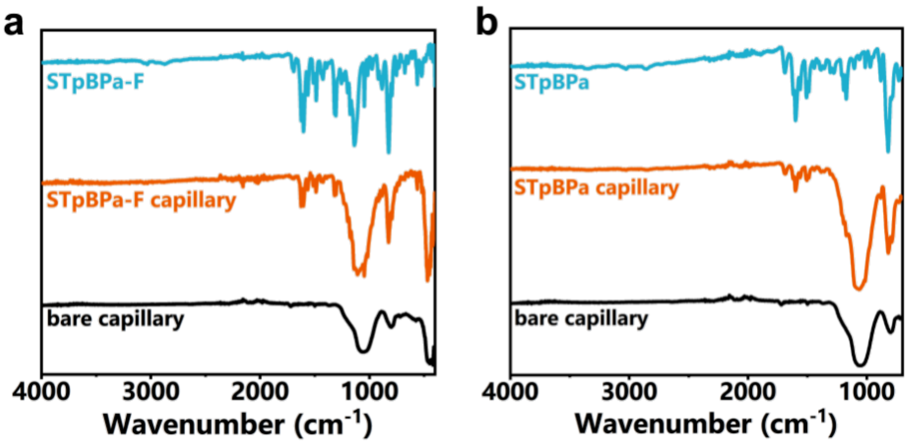

Supplementary Fig. S19 (a) FT-IR spectra of bare capillary, STpBPa-F capillary and STpBPa-F. (b) FT-IR spectra of bare capillary, STpBPa capillary and STpBPa.

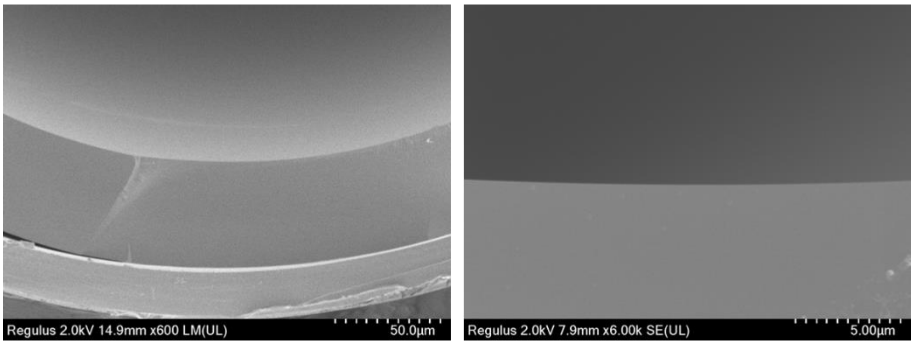

Supplementary Fig. S20 Cross-sectional SEM images of bare capillary column.

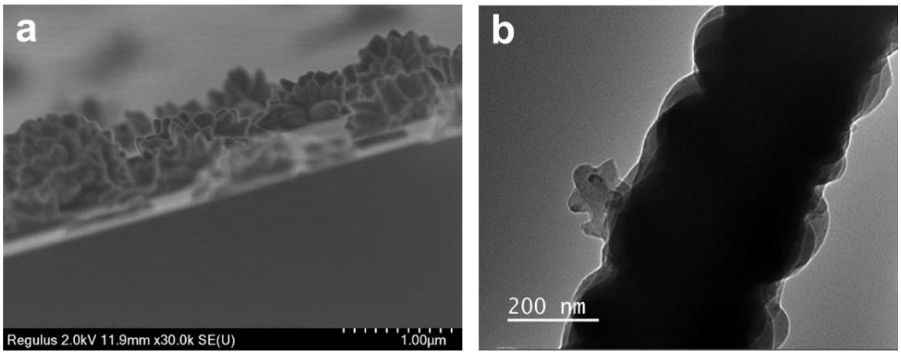

Supplementary Fig. S21 (a) Cross-sectional SEM images of STpBPa-F-bonded capillary column. (b) TEM images of STpBPa-F on capillary column.

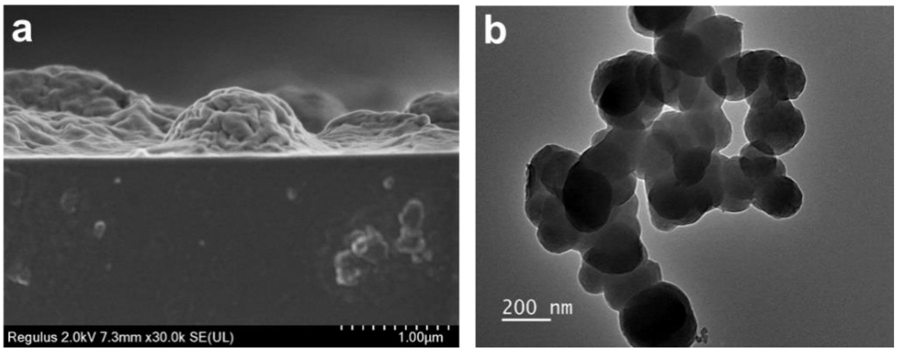

Supplementary Fig. S22 (a) Cross-sectional SEM images of STpBPa capillary column. (b) TEM images of STpBPa on capillary column.

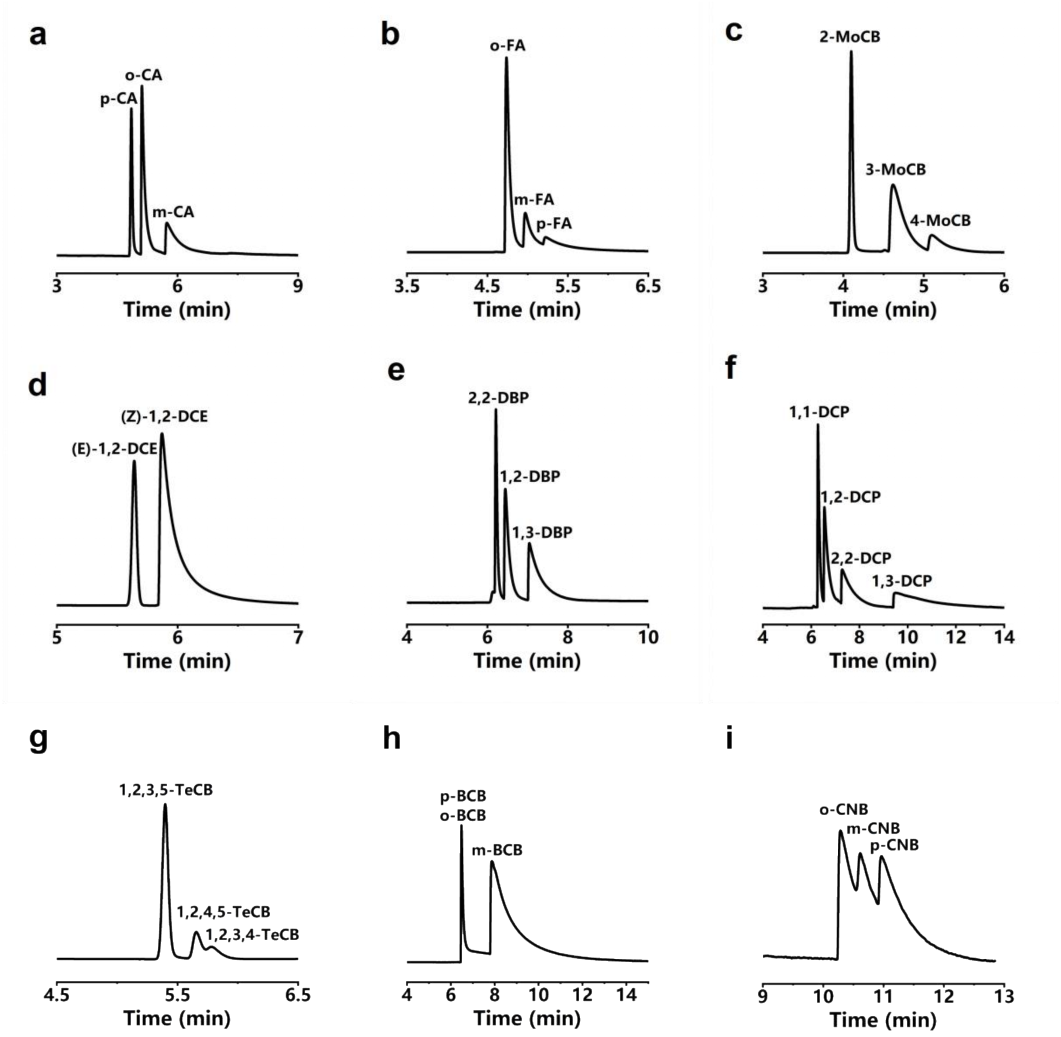

Supplementary Fig. S23 Chromatograms of isomers on the STpBPa-F bonded capillary column. (a) CA (140 °C, 1.2 mL min^{-1} of N_2). (b) FA (140 °C, 1.2 mL min^{-1} of N_2). (c) MoCB (150 °C, 1.5 mL min^{-1} of N_2). (d) 1,2-DCE (80 °C, 1.2 mL min^{-1} of N_2). (e) DBP (80 °C, 1 mL min^{-1} of N_2). (f) DCP (40 °C, 1.2 mL min^{-1} of N_2). (g) TeCB (150 °C, 1.2 mL min^{-1} of N_2). (h) BCB (80 °C, 1.2 mL min^{-1} of N_2). (i) CNB (120 °C, 1 mL min^{-1} of N_2). Separation conditions were optimized to achieve the best separation of the isomers

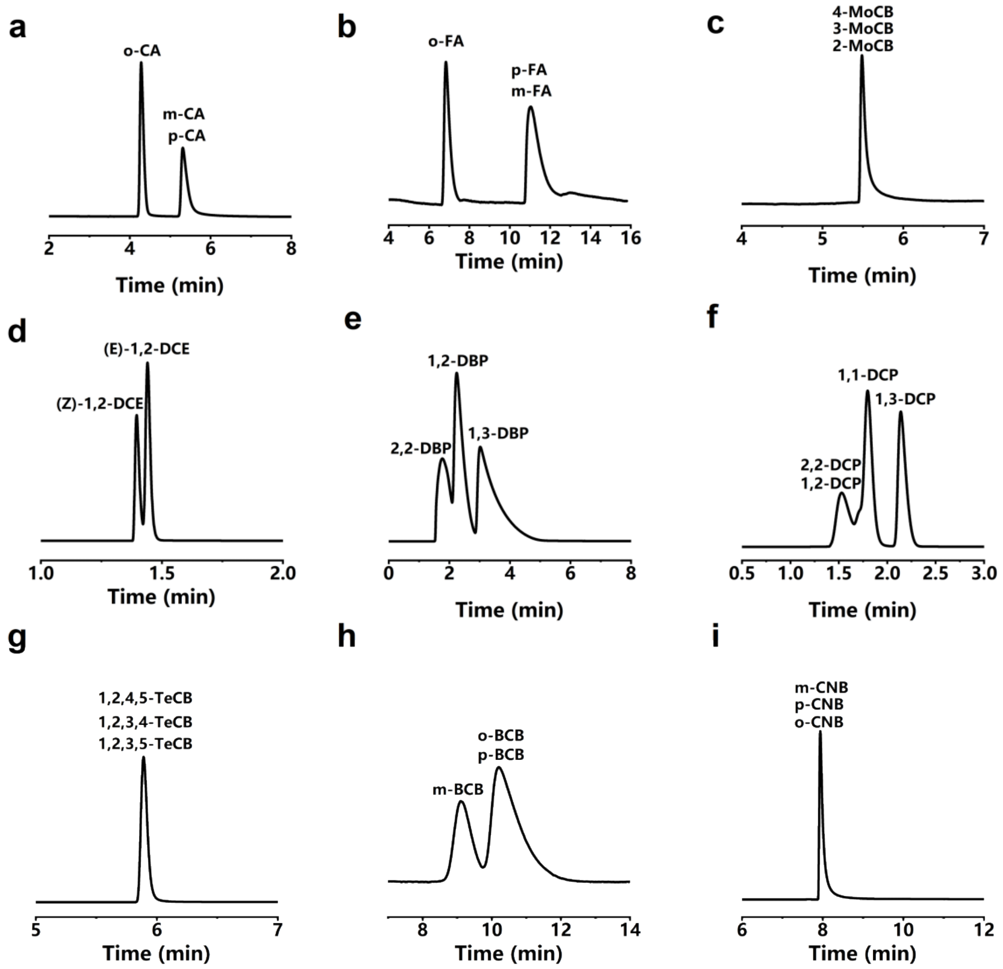

Supplementary Fig. S24 Chromatograms of isomers on the STpBPa bonded capillary column. (a) CA (200 °C, 1.5 mL min^{-1} of N_2). (b) FA (140 °C, 4 mL min^{-1} of N_2). (c) MoCB (200 °C, 1.5 mL min^{-1} of N_2). (d) 1,2-DCE (140 °C, 5 mL min^{-1} of N_2). (e) DBP (170 °C, 1.5 mL min^{-1} of N_2). (f) DCP (100 °C, 5 mL min^{-1} of N_2). (g) TeCB (120 °C, 1.2 mL min^{-1} of N_2). (h) BCB (150 °C, 4 mL min^{-1} of N_2). (i) CNB (100 °C, 1 mL min^{-1} of N_2). Separation conditions were optimized to achieve the best separation of the isomers.

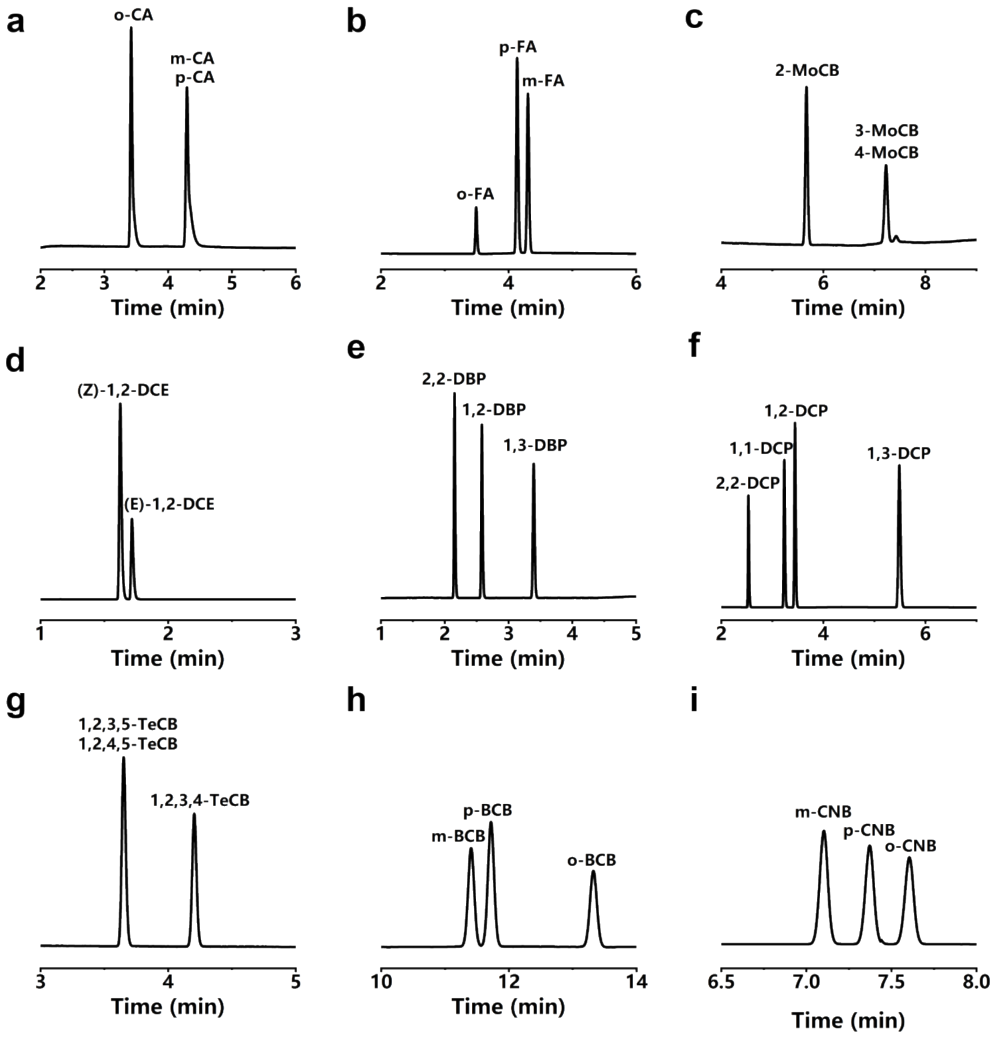

Supplementary Fig. S25 Chromatograms of isomers on the HP-5 commodity column. (a) CA (120 °C, 1.5 mL min^{-1} of N_2). (b) FA (90 °C, 1.5 mL min^{-1} of N_2). (c) MoCB (150 °C, 1.5 mL min^{-1} of N_2). (d) 1,2-DCE (90 °C, 1.5 mL min^{-1} of N_2). (e) DBP (90 °C, 1.5 mL min^{-1} of N_2). (f) DCP (40 °C, 1.5 mL min^{-1} of N_2). (g) TeCB (150 °C, 1.5 mL min^{-1} of N_2). (h) BCB (80 °C, 1.2 mL min^{-1} of N_2). (i) CNB (120 °C, 1 mL min^{-1} of N_2). Separation conditions were optimized to achieve the best separation of the isomers.

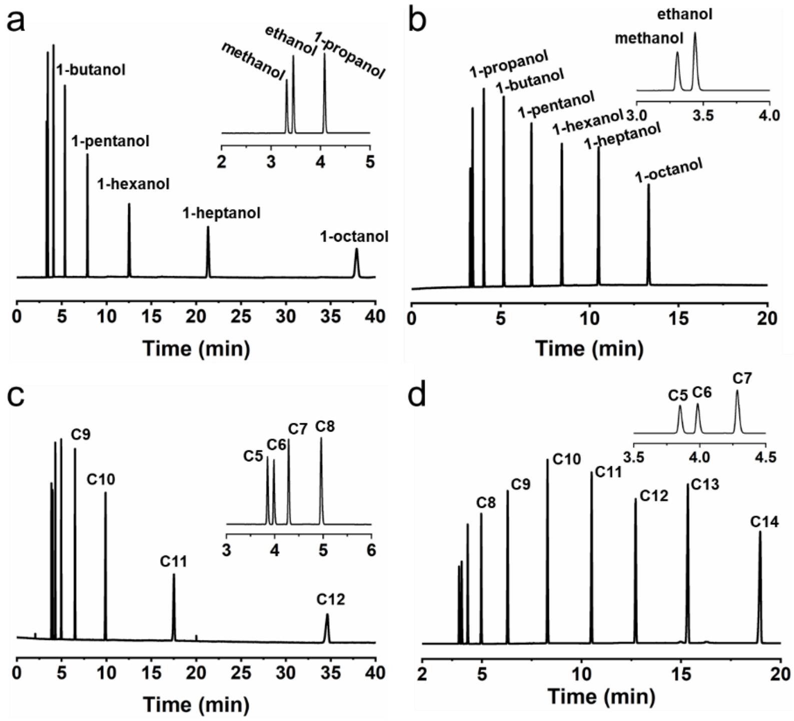

Supplementary Fig. S26 Chromatograms of homologs on HTpBPa-F column. (a) n-alcohols: methanol to 1-octanol (80 °C, 2.4 mL min^{-1} of N_2). (b) n-alcohols: methanol to 1-octanol (80 °C to 120 °C at 10 °C min^{-1}, 2.4 mL min^{-1} of N_2). (c) n-alkanes: n-pentane to n-dodecane (40 °C, 2 mL min^{-1} of N_2). (d) n-alkanes: n-pentane to n-tetradecane (40 °C to 100°C at 10 °C min^{-1}, 2 mL min^{-1} of N_2).

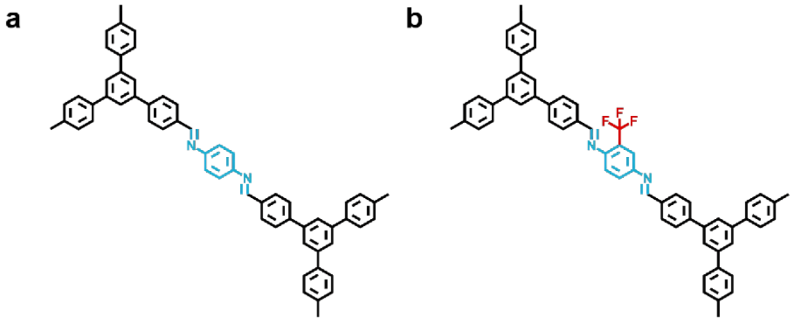

Supplementary Fig. S27 Chemical structure of unit cells representing STpBPa-F and STpBPa for the DFT simulations.

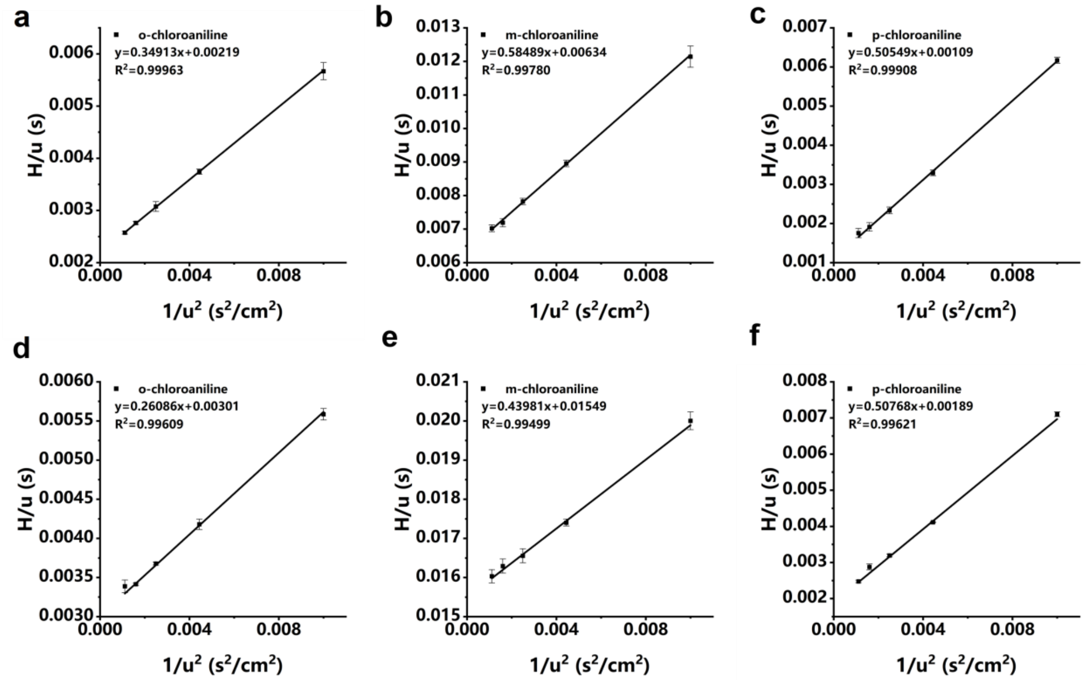

Supplementary Fig. S28 Plots of H/u against $1/u^2$ for three CA isomers on HTpBPa-F (a, b, c) and STpBPa-F columns (d, e, f).

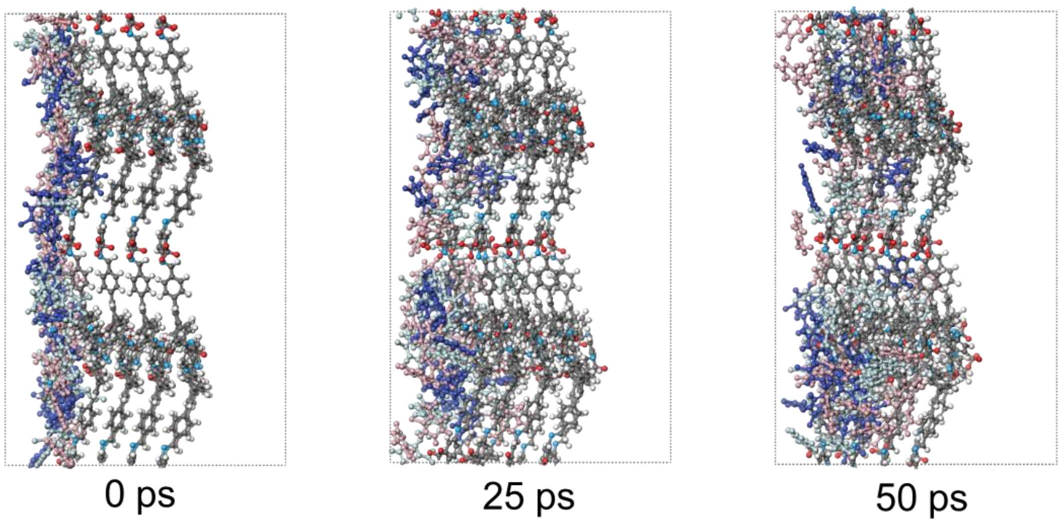

Supplementary Fig. S29 Snapshot images of the MDS process for CA isomers in STpBPa-F.

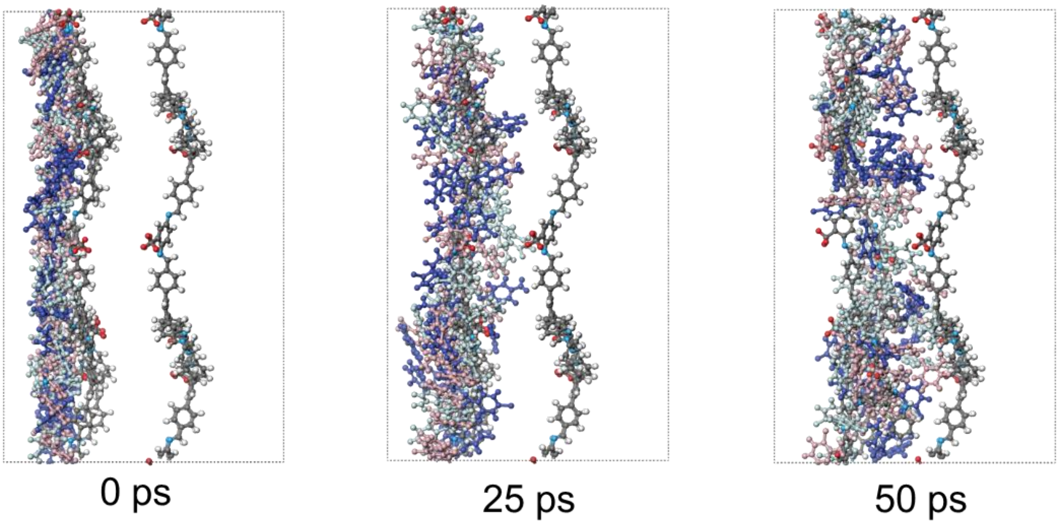

Supplementary Fig. S30 Snapshot images of the MDS process for CA isomers in HTpBPa-F.

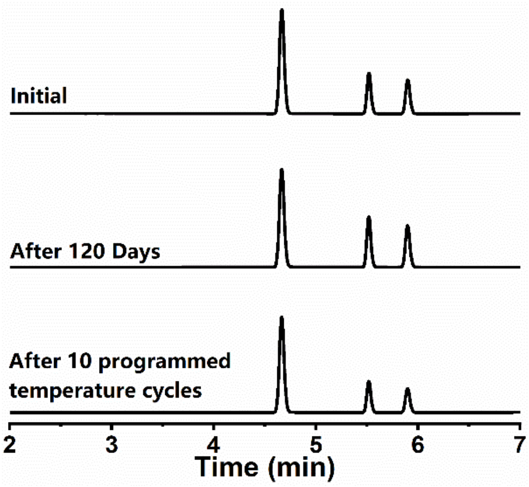

Supplementary Fig. S31 Chromatograms of CA isomers on the same HTpBPa-F column in different conditions (programmed temperature: from 50°C to 300°C at 2°C min^{-1}, keep 300°C for 120 min).

Supplementary Table S1 Fractional main atomic coordinates for the unit cell of HTpBPa-F after Pawley refinement.

Space group symmetry P1<br>a = b = 35.9927 Å, c = 3.7464 Å, α = β = 90° and γ = 120°			
Atom	x	y	z
C1	1.48948	0.53872	0.77005
C2	1.47653	0.49496	0.67971
C3	1.5016	0.47756	0.72187
C4	1.53984	0.50306	0.86007
C5	1.55289	0.54681	0.95997
C6	1.52725	0.56401	0.9136
N7	1.46512	0.55856	0.72562
N8	1.56656	0.48595	0.87164
C9	1.55585	0.44544	0.94279
C10	1.58398	0.42897	0.90743
C11	1.43368	0.5438	0.5354
C12	1.41065	0.56678	0.51427
C13	1.57284	0.38661	1.02165
C14	1.59734	0.36869	0.96282
C15	1.63332	0.39257	0.78451
C16	1.64495	0.43556	0.67894
C17	1.62063	0.45366	0.74118
C18	1.42395	0.60785	0.66734
C19	1.40146	0.62884	0.64238
C20	1.3653	0.60926	0.46273
C21	1.35221	0.56815	0.30986
C22	1.37458	0.54714	0.3363
C23	1.65739	0.37187	0.69097
C24	1.34148	0.63165	0.42908
C25	1.59342	0.57507	1.1202
F26	1.60319	0.55002	1.32423
F27	1.62246	0.59484	0.8767
F28	1.59341	0.60775	1.31512
C29	1.63845	0.32725	0.59994
C30	1.66055	0.30756	0.49732
C31	1.70217	0.33329	0.48269
C32	1.72178	0.37765	0.57883
C33	1.6992	0.39671	0.68304
C34	1.36032	0.67759	0.39542
C35	1.33789	0.6987	0.35611
C36	1.2962	0.67316	0.34237
C37	1.27678	0.62741	0.38149
C38	1.29965	0.60701	0.42742

C39	1.76589	0.40376	0.57028
C40	1.64029	0.25994	0.41404
C41	1.35766	0.74741	0.3398
C42	1.23262	0.60108	0.37558
C43	1.78861	0.38523	0.6829
C44	1.83008	0.40888	0.66224
C45	1.84971	0.45176	0.53131
C46	1.82716	0.47072	0.42523
C47	1.78557	0.44686	0.4431
C48	1.6032	0.23921	0.24917
C49	1.58321	0.19394	0.19044
C50	1.60012	0.16858	0.29235
C51	1.63762	0.18931	0.44719
C52	1.65737	0.23445	0.51005
C53	1.39414	0.77239	0.17053
C54	1.41286	0.81828	0.16469
C55	1.39545	0.84	0.32837
C56	1.359	0.81513	0.4951
C57	1.34032	0.76939	0.50125
C58	1.20968	0.61682	0.53272
C59	1.16812	0.59243	0.52287
C60	1.14865	0.55177	0.35549
C61	1.17148	0.53588	0.20017
C62	1.21306	0.56025	0.2103
C63	1.8935	0.47683	0.50453
C64	1.57857	0.12047	0.25066
C65	1.415	0.88834	0.33768
C66	1.1048	0.52582	0.33622
N67	1.91389	0.45676	0.5248
N68	1.54033	0.09926	0.20187
C69	1.95647	0.47717	0.51101
C70	1.51591	0.05239	0.17917
C71	1.9741	0.45314	0.38741
C72	1.98083	0.51991	0.63605
C73	1.47615	0.03174	0.2958
C74	1.53093	0.02679	0.03997
C75	1.94876	0.40719	0.24557
F76	1.93137	0.3767	0.49947
F77	1.91886	0.40531	0.04549
F78	1.97165	0.39524	0.05334
N79	1.44694	0.91282	0.16225
N80	1.08289	0.53814	0.49868
C81	1.46882	0.96006	0.15886
C82	1.04013	0.5163	0.50203
C83	1.50756	0.9811	0.03122

C84	1.45283	0.98561	0.28046
C85	1.02218	0.53915	0.63053
C86	1.01593	0.47296	0.38682
C87	1.42221	0.96989	0.36224
F88	0.41413	0.93701	0.4435
F89	0.3994	0.96681	0.17437
F90	0.41929	0.98848	0.55916

Supplementary Table S2 Fractional main atomic coordinates for the unit cell of TpBPa after Pawley refinement.

Space group symmetry P6<br>a = b = 35.5650 Å, c = 3.6275 Å, α = β = 90° and γ = 120°			
Atom	x	y	z
C1	0.3792	1.68402	0.50000
C2	0.36221	1.71313	0.50000
C3	0.39106	1.75954	0.50000
C4	0.43376	1.7767	0.50000
C5	0.4598	1.81873	0.50000
C6	0.4443	1.84517	0.50000
C7	0.40218	1.82888	0.50000
C8	0.37601	1.78688	0.50000
C9	0.4724	1.88944	0.50000
N10	0.45789	1.91558	0.50000
C11	0.47929	1.95687	0.50000
C12	0.52197	1.9794	0.50000
C13	0.45659	1.97741	0.50000
H14	0.412	1.69643	0.50000
H15	0.44823	1.75864	0.50000
H16	0.4923	1.83062	0.50000
H17	0.38933	1.84867	0.50000
H18	0.34418	1.777	0.50000
H19	0.50456	1.90015	0.50000
H20	0.54008	1.96486	0.50000
H21	0.42287	1.96016	0.50000

Supplementary Table S3 Resolution of the isomers on the HTpBPa-F, STpBPa-F, STpBPa bonded capillary column and commercial HP-5 column.

Isomer	Resolution (mean ± s, n=3)			
	HTpBPa-F	STpBPa-F	STpBPa	HP-5
Chloroaniline	/	/	/	/
	8.2 ± 0.1	2.7 ± 0.1	4.5 ± 0.1	13.2 ± 0.1
	2.6 ± 0.1	2.5 ± 0.1	0	0
Fluoroaniline	/	/	/	/
	9.9 ± 0.1	2.0 ± 0.1	5.0 ± 0.1	11.2 ± 0.1
	4.9 ± 0.1	1.4 ± 0.1	0	2.7 ± 0.1
Chlorobiphenyl	/	/	/	/
	15.7 ± 0.1	2.7 ± 0.1	0	14.7 ± 0.1
	12.5 ± 0.1	1.5 ± 0.1	0	1.5 ± 0.1
1,2-dichloroethylene	/	/	/	/
	3.3 ± 0.1	2.0 ± 0.1	1.2 ± 0.1	2.6 ± 0.1
Dibromopropane	/	/	/	/
	2.9 ± 0.1	1.8 ± 0.1	0.8 ± 0.1	11.0 ± 0.1
	4.7 ± 0.1	1.8 ± 0.1	1.0 ± 0.1	17.1 ± 0.1
Dichloropropane	/	/	/	/
	6.5 ± 0.1	1.3 ± 0.1	0	16.0 ± 0.1
	2.3 ± 0.1	1.3 ± 0.1	1.3 ± 0.1	4.2 ± 0.1
	4.6 ± 0.1	1.5 ± 0.1	2.2 ± 0.1	30.5 ± 0.3
Tetrachlorobenzene	/	/	/	/
	28.0 ± 0.4	2.2 ± 0.1	0	0
	13.4 ± 0.2	0.9 ± 0.1	0	8.8 ± 0.1
Bromochlorobenzene	/	/	/	/
	3.1 ± 0.1	0	0.9 ± 0.1	1.7 ± 0.1
	8.2 ± 0.1	1.8 ± 0.1	0	8.2 ± 0.1
Chloronitrobenzene	/	/	/	/
	3.0 ± 0.1	1.2 ± 0.1	0	2.9 ± 0.1
	5.3 ± 0.1	1.0 ± 0.1	0	2.4 ± 0.1

$R(2/1) = 2(t_2-t_1) / (W_1+W_2)$, where t is retention time, W is peak width.

The footnotes 1 and 2 refer to the first and second peaks of the two adjacent isomers, respectively.

Supplementary Table S4 Theoretical plate number (N)of the isomers on the HTpBPa-F, STpBPa-F, STpBPa bonded capillary column and commercial HP-5 column.

Isomer		N (plates m^{-1}) (mean ± s, n=3)			
		HTpBPa-F	STpBPa-F	STpBPa	HP-5
Chloroaniline	*p-*	1508 ± 34	1535 ± 123	196 ± 9	1786 ± 49
	o-	2738 ± 70	1074 ± 110	320 ± 22	1797 ± 62
	m-	2716 ± 64	330 ± 49	196 ± 9	1786 ± 49
Fluoroaniline	*o-*	2895 ± 80	1520 ± 122	50 ± 1	2250 ± 83
	m-	2997 ± 77	490 ± 11	101 ± 3	2455 ± 75
	p-	3007 ± 70	365 ± 5	50 ± 1	2351 ± 75
Chlorobiphenyl	*2-*	1405 ± 35	2236 ± 137	1386 ± 53	1963 ± 72
	3-	2653 ± 83	143 ± 5	1386 ± 53	1861 ± 52
	4-	2299 ± 47	164 ± 5	1386 ± 53	1552 ± 39
1,2-Dichloroethylene	*(e)-*	3360 ± 102	2991 ± 162	788 ± 25	1312 ± 69
	(z)-	2540 ± 52	600 ± 12	768 ± 22	1178 ± 65
Dibromopropane	*2,2-*	2452 ± 54	2703 ± 165	12 ± 1	1825 ± 103
	1,2-	2533 ± 63	631 ± 26	19 ± 1	2022 ± 100
	1,3-	2692 ± 86	154 ± 2	13 ± 1	2118 ± 84
Dichloropropane	*1,1-*	1231 ± 29	1372 ± 66	62 ± 7	2339 ± 140
	1,2-	1161 ± 22	312 ± 6	16 ± 1	2323 ± 99
	2,2-	1162 ± 13	58 ± 1	16 ± 1	2109 ± 114
	1,3-	1137 ± 15	12 ± 1	101 ± 6	2378 ± 56
Tetrachlorobenzene	*1,2,3,5-*	7012 ± 255	1671 ± 99	1841 ± 64	1971 ± 64
	1,2,4,5-	7389 ± 240	869 ± 52	1841 ± 64	1971 ± 64
	1,2,3,4-	5620 ± 160	1036 ± 92	1841 ± 64	2184 ± 67
Bromochlorobenzene	*p-*	3663 ± 40	1123 ± 64	47 ± 1	2108 ± 23
	o-	3634 ± 83	1123 ± 64	28 ± 1	2097 ± 34
	m-	3627 ± 61	20 ± 1	28 ± 1	1998 ± 21
Chloronitrobenzene	*o-*	3281 ± 127	763 ± 26	1460 ± 69	3138 ± 63
	m-	3249 ± 68	853 ± 12	1460 ± 69	3117 ± 67
	p-	3258 ± 71	337 ± 8	1460 ± 69	3162 ± 65

Supplementary Table S5 Molecular size of halogenated isomers.

Isomer		Three-dimensional dimensions a × b × c (Å)
Chloroaniline	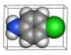	9.427 × 6.568 × 3.560
	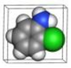	8.512 × 7.803 × 3.560
	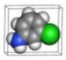	8.993 × 7.646 × 3.560
Fluoroaniline	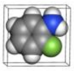	7.793 × 7.237 × 3.201
	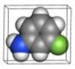	8.266 × 7.237 × 3.201
	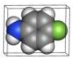	8.639 × 6.564 × 3.201
Chlorobiphenyl	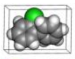	11.531 × 8.005 × 3.856
	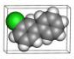	11.735 × 8.305 × 3.789
	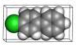	12.831 × 6.513 × 3.584
1,2-Dichloroethylene	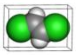	7.866 × 5.257 × 3.560
	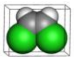	6.616 × 5.322 × 3.560
Dibromopropane	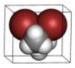	7.022 × 5.668 × 6.503
	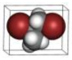	8.028 × 6.258 × 4.916
	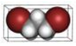	9.471 × 4.776 × 4.017
Dichloropropane	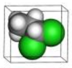	7.348 × 6.525 × 5.080
	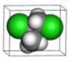	7.492 × 6.158 × 4.863
	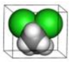	6.480 × 5.380 × 6.491
	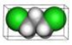	8.979 × 4.521 × 4.018
Tetrachlorobenzene	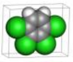	9.788×7.737×3.560
	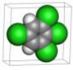	9.832×8.993×3.560
	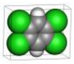	8.995×7.251×3.560
Bromochlorobenzene	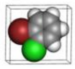	8.783×7.879×3.840
	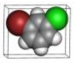	9.238×7.270×3.840
	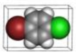	10.125×6.570×3.840
Chloronitrobenzene	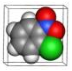	8.630 × 7.547 × 3.560
	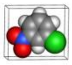	9.315 × 7.841 × 3.560
	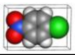	9.621 × 6.521 × 3.560

The molecular size were calculated using the online tool[7.] (https://jerkwin.github.io/2016/06/24/%E5%88%86%E5%AD%90%E5%B0%BA%E5%AF%B8%E5%A4%A7%E5%B0%8F%E7%9A%84%E8%AE%A1%E7%AE%97/)

Supplementary Table S6 Partition coefficient (*K*) and partition coefficient difference (Δ*K*) of the isomers on the HTpBPa-F bonded capillary column.

Isomer		*K* (mean ± s, n=3)	*ΔK* (mean ± s, n=3)
Chloroaniline	*p-*	70.9 ± 0.1	/
	o-	122.0 ± 0.2	51.3 ± 0.3
	m-	137.1 ± 0.2	14.9 ± 0.1
Fluoroaniline	*o-*	37.3 ± 0.1	/
	m-	80.6 ± 0.1	43.3 ± 0.1
	p-	104.0 ± 0.1	23.4 ± 0.1
Chlorobiphenyl	*2-*	35.9 ± 0.6	/
	3-	125.9 ± 1.2	90.0 ± 0.6
	4-	205.3 ± 0.6	79.4 ± 0.6
1,2-Dichloroethylene	*(e)-*	77.6 ± 2.7	/
	(z)-	90.2 ± 1.1	13.8 ± 2.7
Dibromopropane	*2,2-*	54.0 ± 2.0	/
	1,2-	65.3 ± 0.7	12.5 ± 1.8
	1,3-	87.9 ± 1.8	23.2 ± 0.1
Dichloropropane	*1,1-*	59.9 ± 2.4	/
	1,2-	105.9 ± 0.6	46.4 ± 2.2
	2,2-	124.0 ± 1.1	18.0 ± 0.7
	1,3-	166.9 ± 2.4	42.4 ± 3.0
Tetrachlorobenzene	*1,2,3,5-*	119.8 ± 1.4	/
	1,2,4,5-	221.1 ± 3.3	100.9 ± 2.7
	1,2,3,4-	287.5 ± 1.3	66.9 ± 3.3
Bromochlorobenzene	*p-*	331.0 ± 3.2	/
	o-	354.2 ± 1.7	22.0 ± 0.7
	m-	417.3 ± 0.8	63.4 ± 2.2
Chloronitrobenzene	*o-*	100.8 ± 0.3	/
	m-	114.8 ± 0.2	14.2 ± 0.1
	p-	142.3 ± 0.6	27.2 ± 0.2

Supplementary Table S7 Partition coefficient (*K*) and partition coefficient difference (Δ*K*) of the isomers on the STpBPa-F bonded capillary column.

Isomer		*K* (mean ± s, n=3)	*ΔK* (mean ± s, n=3)
Chloroaniline	*p-*	25.7 ± 0.5	/
	o-	41.5 ± 0.2	15.6 ± 0.2
	m-	78.3 ± 0.1	36.9 ± 0.2
Fluoroaniline	*o-*	27.5 ± 0.2	/
	m-	38.9 ± 0.2	11.3 ± 0.1
	p-	51.8 ± 0.9	12.6 ± 0.8
Chlorobiphenyl	*2-*	47.9 ± 0.4	/
	3-	79.3 ± 0.3	31.4 ± 0.1
	4-	97.9 ± 0.6	18.6 ± 0.4
1,2-Dichloroethylene	*(e)-*	34.6 ± 0.1	/
	(z)-	44.1 ± 0.1	9.6 ± 0.1
Dibromopropane	*2,2-*	15.9 ± 0.2	/
	1,2-	23.8 ± 0.5	8.0 ± 0.3
	1,3-	44.6 ± 0.5	20.7 ± 0.2
Dichloropropane	*1,1-*	30.4 ± 0.7	/
	1,2-	40.3 ± 0.7	9.9 ± 0.1
	2,2-	57.5 ± 0.4	17.1 ± 0.3
	1,3-	89.2 ± 0.5	31.8 ± 0.2
Tetrachlorobenzene	*1,2,3,5-*	65.0 ± 0.6	/
	1,2,4,5-	77.6 ± 0.8	12.7 ± 0.1
	1,2,3,4-	82.0 ± 2.1	5.3 ± 1.5
Bromochlorobenzene	*p-*	68.0 ± 1.5	/
	o-	68.0 ± 1.5	0
	m-	94.7 ± 0.7	26.4 ± 1.3
Chloronitrobenzene	*o-*	194.8 ± 0.3	/
	m-	207.2 ± 0.3	12.6 ± 0.1
	p-	219.9 ± 1.4	13.4 ± 0.3

Supplementary Table S8 Partition coefficient (K) and partition coefficient difference (ΔK) of the isomers on the STpBPa bonded capillary column.

Isomer		K (mean ± s, n=3)	ΔK (mean ± s, n=3)
Chloroaniline	*o-*	60.1 ± 1.0	/
	m-	92.6 ± 0.3	32.2 ± 1.0
	p-	92.6 ± 0.3	0
Fluoroaniline	*o-*	79.8 ± 0.2	/
	p-	141.3 ± 0.5	61.6 ± 0.1
	m-	141.3 ± 0.5	0
Chlorobiphenyl	*4-*	24.5 ± 0.1	/
	3-	24.5 ± 0.1	0
	2-	24.5 ± 0.1	0
1,2-Dichloroethylene	*(z)-*	18.6 ± 0.8	/
	(e)-	21.0 ± 1.5	2.9 ± 0.5
Dibromopropane	*2,2-*	16.9 ± 0.3	/
	1,2-	29.1 ± 0.9	12.6 ± 0.2
	1,3-	50.4 ± 0.3	20.9 ± 0.3
Dichloropropane	*2,2-*	24.5 ± 0.8	/
	1,2-	24.5 ± 0.8	0
	1,1-	41.5 ± 0.6	16.8 ± 0.3
	1,3-	62.1 ± 1.6	21.2 ± 0.1
Tetrachlorobenzene	*1,2,4,5-*	24.8 ± 0.1	/
	1,2,3,4-	24.8 ± 0.1	0
	1,2,3,5-	24.8 ± 0.1	0
Bromochlorobenzene	*m-*	289.2 ± 0.5	/
	o-	329.7 ± 0.8	40.7 ± 0.8
	p-	329.7 ± 0.8	0
Chloronitrobenzene	*m-*	32.3 ± 0.1	/
	p-	32.3 ± 0.1	0
	o-	32.3 ± 0.1	0

Supplementary Table S9 Thermodynamic parameters for the separation of halogenated isomers on a HTpBPa-F bonded capillary column.

Isomer		ΔH (KJ mol^{-1})	ΔS (J mol^{-1} K^{-1})	R^2
Chloroaniline	*p-*	-17.9 ± 2.3	-11.5 ± 1.2	0.9982
	o-	-24.0 ± 1.2	-25.0 ± 0.7	0.9997
	m-	-38.2 ± 8.5	-55.9 ± 4.6	0.9946
Fluoroaniline	*o-*	-18.7 ± 0.9	-13.6 ± 0.5	0.9998
	m-	-24.6 ± 1.2	-26.3 ± 0.6	0.9998
	p-	-42.9 ± 6.5	-67.2 ± 3.5	0.9975
Chlorobiphenyl	*2-*	-16.2 ± 1.2	-8.8 ± 0.7	0.9994
	3-	-16.2 ± 1.3	-6.8 ± 0.7	0.9993
	4-	-16.7 ± 0.9	-5.0 ± 0.5	0.9997
1,2-Dichloroethylene	*(e)-*	-13.7 ± 0.7	-10.6 ± 0.5	0.9997
	(z)-	-12.1 ± 0.2	-7.0 ± 0.1	0.9999
Dibromopropane	*2,2-*	-22.0 ± 5.9	-29.4 ± 3.7	0.9924
	1,2-	-25.5 ± 3.8	-37.3 ± 2.4	0.9976
	1,3-	-32.1 ± 3.9	-53.5 ± 2.5	0.9984
Dichloropropane	*1,1-*	-15.9 ± 3.0	-13.6 ± 2.1	0.9963
	1,2-	-15.4 ± 1.1	-11.4 ± 0.8	0.9995
	2,2-	-19.1 ± 3.4	-22.3 ± 2.4	0.9967
	1,3-	-27.0 ± 4.9	-43.2 ± 3.5	0.9964
Tetrachlorobenzene	*1,2,3,5-*	-27.1 ± 1.7	-26.1 ± 1.0	0.9996
	1,2,4,5-	-24.9 ± 1.7	-19.8 ± 1.0	0.9995
	1,2,3,4-	-19.9 ± 1.0	-5.4 ± 0.6	0.9997
Bromochlorobenzene	*p-*	-18.0 ± 1.2	-8.4 ± 0.8	0.9995
	o-	-18.9 ± 1.3	-10.4 ± 0.8	0.9995
	m-	-20.5 ± 1.2	-13.7 ± 0.8	0.9996
Chloronitrobenzene	*o-*	-26.2 ± 1.9	-30.4 ± 1.2	0.9994
	m-	-25.6 ± 4.1	-28.0 ± 2.5	0.9973
	p-	-26.9 ± 3.9	-29.9 ± 2.4	0.9977

Supplementary Table S10 Theoretical plate height (*H*) of the isomers on the HTpBPa-F, STpBPa-F and STpBPa bonded capillary column.

Isomer		*H* (mm) (mean ± s, n=3)		
		HTpBPa-F	STpBPa-F	STpBPa
Chloroaniline	*p*-	0.66 ± 0.01	0.65 ± 0.05	5.12 ± 0.23
	o-	0.37 ± 0.01	0.94 ± 0.10	3.13 ± 0.21
	m-	0.37 ± 0.01	3.01 ± 0.45	5.12 ± 0.23
Fluoroaniline	*o*-	0.35 ± 0.01	0.66 ± 0.05	9.95 ± 0.31
	m-	0.33 ± 0.01	2.04 ± 0.05	19.99 ± 0.34
	p-	0.33 ± 0.01	2.74 ± 0.04	19.99 ± 0.34
Chlorobiphenyl	*2*-	0.71 ± 0.02	0.45 ± 0.03	0.72 ± 0.03
	3-	0.38 ± 0.01	6.98 ± 0.24	0.72 ± 0.03
	4-	0.44 ± 0.01	6.11 ± 0.20	0.72 ± 0.03
1,2-Dichloroethylene	*(e)*-	0.30 ± 0.01	0.33 ± 0.02	1.27 ± 0.04
	(z)-	0.39 ± 0.01	1.67 ± 0.03	1.30 ± 0.04
Dibromopropane	*2,2*-	0.41 ± 0.01	0.37 ± 0.02	81.37 ± 3.05
	1,2-	0.39 ± 0.01	1.59 ± 0.06	52.60 ± 1.44
	1,3-	0.37 ± 0.01	6.49 ± 0.10	77.22 ± 4.26
Dichloropropane	*1,1*-	0.81 ± 0.02	0.73 ± 0.03	16.18 ± 1.77
	1,2-	0.86 ± 0.02	3.21 ± 0.06	64.07 ± 3.01
	2,2-	0.86 ± 0.01	17.21 ± 0.21	64.07 ± 3.01
	1,3-	0.88 ± 0.01	81.61 ± 2.65	9.91 ± 0.56
Tetrachlorobenzene	*1,2,3,5*-	0.14 ± 0.01	0.60 ± 0.03	0.54 ± 0.02
	1,2,4,5-	0.14 ± 0.01	1.15 ± 0.07	0.54 ± 0.02
	1,2,3,4-	0.18 ± 0.01	0.97 ± 0.09	0.54 ± 0.02
Bromochlorobenzene	*p*-	0.27 ± 0.01	0.89 ± 0.05	36.18 ± 1.44
	o-	0.28 ± 0.01	0.89 ± 0.05	36.18 ± 1.44
	m-	0.28 ± 0.01	49.47 ± 0.78	21.33 ± 0.54
Chloronitrobenzene	*o*-	0.31 ± 0.01	1.31 ± 0.04	0.69 ± 0.03
	m-	0.31 ± 0.01	1.17 ± 0.02	0.69 ± 0.03
	p-	0.31 ± 0.01	2.97 ± 0.07	0.69 ± 0.03

Supplementary Table S11 Van Deemter coefficients of CA isomers on HTpBPa-F bonded capillary column and STpBPa-F bonded capillary column.

Isomer	Van Deemter coefficient	HTpBPa-F capillary	STpBPa-F capillary
o-CA	*B* ($mm^2\ s^{-1}$)	34.9	26.1
	C (s)	$2.19*10^{-3}$	$3.01*10^{-3}$
m-CA	*B* ($mm^2\ s^{-1}$)	58.5	44.0
	C (s)	$6.34*10^{-3}$	$15.5*10^{-3}$
p-CA	*B* ($mm^2\ s^{-1}$)	50.5	50.8
	C (s)	$1.09*10^{-3}$	$1.89*10^{-3}$

Supplementary Table S12 Comparative evaluation of CA isomers separation performance on the same HTpBPa-F column in different time.

Isomer	Initial (mean ± s, n=3)		After 120 Days		After temperature-programmed	
	N (plates m^{-1})	*k* ($\times 10^{-3}$)	Reduction in N (%)	Reduction in k' (%)	Reduction in N (%)	Reduction in k' (%)
p-CA	1485 ± 26	129.0 ± 0.1	0.4	0.2	4.1	0.5
o-CA	2863 ± 74	263.5 ± 0.1	2.6	0.1	5.5	0.2
m-CA	2752 ± 65	311.0 ± 0.1	2.4	0.1	5.4	0.1

Supplementary Table S13 Precision for the separation of isomers on the HTpBPa-F capillary columns.

Isomer	Relative standard deviation (%)					
	Run-to-run (n = 8)		Day-to-day (n = 5)		Column-to-column (n = 3)	
	Retention time	peak area	Retention time	peak area	Retention time	peak area
p-CA	0.11	3.0	0.10	6.1	0.11	6.8
o-CA	0.11	3.2	0.11	5.8	0.22	7.4
m-CA	0.11	2.8	0.11	6.1	0.22	7.2

Supplementary References

(1) Kumawat, S.; Natte, K. Iron(II) triflate as a photocatalyst for trifluoromethylation of functionalized arenes under blue LED light: Access to bioactive compounds. *J. Catal.* **2024**, *434*, 10.

(2) Stephens, P. J.; Devlin, F. J.; Ashvar, C. S.; Chabalowski, C. F.; Frisch, M. J. Theoretical calculation of vibrational circular-dichroism spectra. *Faraday Discuss.* **1994**, *99*, 103-119.

(3) Becke, A. D. Density-functional thermochemistry. III. The role of exact exchange. *J. Chem. Phys.* **1993**, *98,* 5648-5652.

(4) Weigend, F.; Ahlrichs, R. Balanced basis sets of split valence, triple zeta valence and quadruple zeta valence quality for h to rn: Design and assessment of accuracy. *Phys. Chem. Chem. Phys.* **2005**, *7,* 3297-3305.

(5) Weigend, F. Accurate coulomb-fitting basis sets for h to rn. *Phys. Chem. Chem. Phys.* **2006**, *8*, 1057-1065.

(6) Columbié-Leyva, R.; Miranda, U.; López-Vivas, A.; Soullard, J.; Kaplan, I. G. Quantum Mechanical Calculations of High-Tc Fe-Superconductors. *J. Quantum Inf. Sci.* **2021**, 11, 84-98.

(7) Mantina, M.; Chamberlin, A. C.; Valero, R.; Cramer, C. J.; Truhlar, D. G.; Consistent van der Waals Radii for the Whole Main Group. *Phys. Chem. A.* **2009**, 113, 5806-5812.
